# The interplay among narcissistic vulnerability, interpersonal sensitivity, and metacognitive integration: A network analysis approach

**DOI:** 10.1192/j.eurpsy.2026.12226

**Published:** 2026-06-16

**Authors:** Matteo Aloi, Aaron Lee Pincus, Antonio Semerari, Ilaria Bucci, Livia Colle, Giuseppe Nicolò, Ilaria Riccardi, Cristina Segura-Garcia, Antonino Carcione

**Affiliations:** 1 Department of Clinical and Experimental Medicine, University of Messina, Messina, Italy; 2 Department of Psychology, University of Pennsylvania, Philadelphia, PA, USA; 3 Third Centre of Cognitive Psychotherapy – Italian School of Cognitive Psychotherapy (SICC), Rome, Italy; 4 Department of Psychology, University of Turin, Turin, Italy; 5 Department of Medical and Surgery Sciences, University of Catanzaro, Catanzaro, Italy; 6 Department of Biotechnological and Applied Clinical Sciences, University of L’Aquila, L’Aquila, Italy; 7 Department of Human Science, “Guglielmo Marconi” University, Rome, Italy

**Keywords:** metacognitive interpersonal treatment, narcissism, network analysis, personality disorders, psychotherapy

## Abstract

**Background:**

Narcissistic Personality Disorder (NPD) involves disturbances in self-regulation, interpersonal functioning, and personality organization. Although traditionally characterized by grandiosity, contemporary models suggest that grandiose self-states coexist with vulnerable features such as shame, emotional dysregulation, and hypersensitivity to rejection. Recent evidence indicates that metacognitive impairments may underlie both grandiose and vulnerable narcissistic presentations; however, no study has examined how metacognition interacts with personality traits and interpersonal difficulties within an integrated system.

**Methods:**

A cross-sectional network analysis was conducted on 287 patients with NPD. Measures included the Metacognition Assessment Interview, the Pathological Narcissism Inventory, the Personality Inventory for DSM-5 (PID-5), the Inventory of Interpersonal Problems, and SCL-90-R Depression. A Gaussian graphical model with LASSO regularization was estimated, and expected influence was used as the primary index of node centrality. Network accuracy and stability were assessed through bootstrapping procedures.

**Results:**

Narcissistic vulnerability was the most central node, followed by interpersonal sensitivity and metacognitive integration. Narcissistic vulnerability showed strong associations with PID-5 Negative Affectivity and Detachment, whereas narcissistic grandiosity was related to PID-5 Antagonism. Metacognitive integration occupied a central position, linking maladaptive traits and interpersonal distress. Network stability indices indicated good reliability.

**Conclusions:**

Findings suggest that narcissistic vulnerability and interpersonal hypersensitivity are central aspects of dysfunction in NPD, whereas metacognitive integration appears closely associated with the organization of psychological processes within the network. Although causal inferences cannot be drawn, the results are consistent with theoretical models underlying Metacognitive Interpersonal Therapy (MIT), supporting the potential relevance of targeting integrative metacognitive capacities in NPD treatment.

## Introduction

Narcissistic personality disorder (NPD) is a multifaceted and heterogeneous personality disorder characterized by persistent disturbances in self-regulation, interpersonal functioning, and personality organization. Across revisions of the *Diagnostic and Statistical Manual of Mental Disorders* (DSM), NPD became defined largely by its more visible grandiose features [1]. These include pervasive attitudes of superiority, entitlement, a strong need for admiration, significant deficits in empathy, and exploitative interpersonal behavior [2]. However, more recent research has underscored the importance of narcissistic vulnerability, a clinically impactful presentation characterized by heightened sensitivity to criticism, profound shame, difficulties with emotion regulation, and an unstable sense of self [3–6]. These two dimensions are not mutually exclusive; rather, individuals with NPD may fluctuate between displays of superiority and feelings of insecurity, and this fluctuation contributes significantly to the clinical complexity of the disorder [7–9].

Recent theoretical models suggest that disturbance in metacognitive functioning, namely, the capacity to recognize, interpret, integrate, and reflect upon mental states [10, 11], may play a foundational role in shaping the expression of pathological narcissism [12]. Metacognition and mentalization are closely related constructs, both referring to the capacity to reflect upon and reason about one’s own and others’ mental states, and both are conceptualized as multidimensional processes [11, 13]. However, important differences can be identified. Mentalization has often been described in terms of dysfunctional modes of reflecting on mental states, particularly hypermentalization (i.e., over-interpretation of mental states) and hypomentalization (i.e., reduced or simplistic understanding of mental states), which have been linked to various forms of psychopathology [14–16]. In contrast, metacognition, as defined by Semerari and colleagues, encompasses a broader set of capacities, including the ability to identify, differentiate, integrate, and reflect upon mental states, as well as to use this knowledge to cope with psychological distress and interpersonal problems (i.e., metacognitive mastery) [17, 18]. Furthermore, unlike mentalization, metacognition does not necessarily presuppose the activation of the attachment system as the primary source of psychopathology [19].

Metacognitive impairments have been linked to identity instability, impaired emotion regulation, and maladaptive interpersonal strategies, processes highly relevant to both narcissistic grandiosity and vulnerability [20]. In particular, reduced metacognitive integration, i.e., the ability to maintain a coherent and unified self-representation across fluctuating internal states and contexts, may exacerbate the instability and self-fragmentation commonly observed in narcissistic vulnerability [19, 21, 22]. On the other hand, impairments in differentiation and decentration may reduce the ability to consider others’ perspectives, thus reinforcing self-centered reasoning and rigid grandiose postures [12].

In this context, interpersonal dysfunction represents a central and well-documented feature of pathological narcissism. Decades of research using an interpersonal theory framework have shown that individuals higher in narcissistic traits tend to exhibit dominant, cold, and conflict-prone interpersonal behaviors, which contribute to relational instability and chronic dissatisfaction [23–25]. These interpersonal difficulties are not only dispositional but also dynamic and context-dependent, arising strongly in moment-to-moment social interactions. Using ecological momentary assessment (EMA), Wright and colleagues showed that people high in pathological narcissism are particularly reactive to perceived interpersonal dominance in others: when they perceive that someone is asserting control or status, they experience increased negative affect and respond with greater quarrelsomeness, hostility, and interpersonal coldness [26]. These reactions seem to serve short-term self-regulatory aims (i.e., protecting self-esteem and reasserting control) but paradoxically intensify interpersonal conflict and erode relationship stability [27]. Notably, both grandiose and vulnerable narcissistic traits contribute to these patterns through different pathways: grandiosity is associated with socially dominant and antagonistic behaviors, whereas vulnerability is linked to heightened interpersonal sensitivity, shame, and a tendency to perceive others as threatening or rejecting [28, 29]. Taken together, this body of evidence suggests that pathological narcissism is maintained through reciprocal interpersonal processes, in which perceived threats trigger maladaptive affective responses and antagonistic behaviors, reinforcing cycles of misunderstanding, rejection, and self-esteem dysregulation.

Complementing this interpersonal and metacognitive perspective, contemporary trait-based models offer a broader framework for understanding the dispositional architecture that underlies these relational and self-regulatory patterns. The Alternative Model for Personality Disorders (AMPD) provides a dimensional framework for understanding the maladaptive personality traits associated with NPD, with its domains capturing broad patterns of emotional, interpersonal, and perceptual dysfunction [30, 31]. Within this model, NPD is defined by impairments in self and interpersonal functioning and by the elevation of two specific maladaptive traits: grandiosity and attention seeking, which constitute the core trait criteria for the diagnosis [2]. Among the broader AMPD domains, Antagonism shows the most consistent association with narcissistic grandiosity and interpersonal exploitation, aligning closely with these diagnostic features [32]. However, a growing body of research documents that narcissistic pathology cannot be fully captured by antagonistic traits alone. Clinically oriented assessments reveal notable associations with Negative Affectivity, reflecting emotional instability and vulnerability, and with Psychoticism, reflecting cognitive-perceptual dysregulation [33]. Furthermore, AMPD level of personality functioning emphasizes the importance of reflective functioning, conceptualized as metacognition or mentalization [14, 34], in sustaining a stable sense of self and promoting adaptive interpersonal relationships. Low levels of metacognition represent negative prognostic factors, increasing vulnerability to interpersonal dysfunction and symptomatic distress. Patients with NPD showed poorer metacognition than patients without PD, comparable to patients with other PDs, and metacognitive difficulties appear to be related to specific aspects of pathological narcissism [12].

This pattern indicates that pathological narcissism reflects a broader and more complex constellation of AMPD traits than grandiosity and attention seeking alone would suggest.

Given the interplay among these metacognitive, personality, interpersonal, and affective domains, traditional statistical approaches may well overlook the dynamic and reciprocal nature of NPD’s psychological architecture. Network analysis provides a promising alternative, reconceptualizing psychopathology as a system of interconnected components rather than as manifestations of latent constructs [35]. This approach models each variable as a node and quantifies the unique associations between nodes while controlling for all others, thus allowing researchers to identify central nodes that exert a disproportionate influence upon the system [36]. Network analysis is particularly suited to NPD because symptoms and psychological processes tend to be structurally interrelated, reciprocally reinforcing each other and forming patterns of co-activation that are concordant with clinically observed feedback loops. In cross-sectional networks, such ’dynamic’ patterns refer to contemporaneous interdependencies among variables, as opposed to temporal or causal processes [35, 36]. For instance, interpersonal rejection may co-occur with heightened narcissistic vulnerability, reduced metacognitive integration, and increased interpersonal sensitivity, forming a theoretically meaningful configuration consistent with clinically observed dynamics [19]. To date, no study has investigated the combined network of metacognition, pathological personality traits, interpersonal difficulties, and narcissistic dimensions in patients with NPD. The interaction among these domains may provide a deeper understanding of the core mechanisms that sustain narcissistic pathology and may indicate new targets for therapeutic intervention, especially in metacognitively oriented treatments like Metacognitive Interpersonal Therapy (MIT; [10]), which has already obtained some evidence of efficacy [37–39].

The aim of the current study is to model the network structure of a clinically diagnosed NPD sample by integrating metacognitive capacities, narcissistic grandiosity and vulnerability, maladaptive personality traits, interpersonal functioning, and depressive symptoms. We apply a Gaussian graphical model with LASSO regularization to identify (1) the most central psychological features in the network, (2) key connections among constructs, and (3) the specific contribution of metacognitive functions to the broader system of narcissistic pathology. This approach may offer a more nuanced depiction of NPD and inform precision targets for psychotherapy.

## Methods

### Participants

This cross-sectional study was carried out with patients consecutively evaluated at a specialized outpatient center for personality disorders (Third Center of Cognitive Psychotherapy, Rome) from July 2021 to May 2025. All participants gave their written informed consent for the use of anonymized clinical data for research purposes during their first visit. To be included in the present study, participants had to meet the DSM-5 [2] diagnostic criteria for NPD and be at least 18 years of age. Further, all participants included in the NPD group reached five or more diagnostic criteria for NPD while scoring below threshold levels for all other personality disorders, in order to ensure a homogeneous diagnostic sample. This approach was adopted to reduce diagnostic heterogeneity and isolate the specific network structure of NPD. Exclusion criteria included lifetime diagnosis of schizophrenia, schizoaffective disorder, and bipolar disorder, as well as substance abuse or dependence within the three months prior to assessment. The criteria also excluded organic mental syndromes, dementia, cognitive impairment, and major neurological disorders. Overall, these procedures were designed to ensure a clinically well-defined and methodologically appropriate sample for network analysis.

### Study sample size

**Figure 1.**
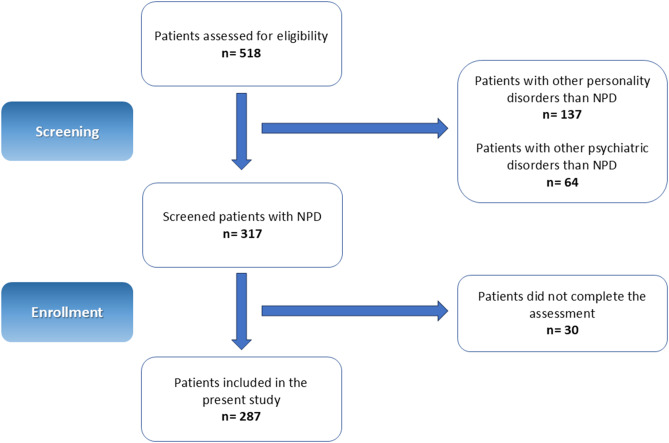
Sample flow-chart. Figure 1. long description.

Of the initial 518 patients assessed, 454 received a diagnosis of a personality disorder, while 64 were diagnosed with other psychiatric conditions and were therefore excluded. Among those with personality disorders, 317 met criteria for NPD. However, 30 of these individuals did not complete the assessments required for inclusion in the present analysis, in order to avoid managing missing data (Figure 1). Thus, the final sample consisted of 287 patients with NPD (146 female, 50.9%) included in the study.

No formal a priori power analysis was conducted. However, the final sample size (*N* = 287) is consistent with current methodological recommendations for psychological network analysis, which emphasize the use of sufficiently large samples to ensure stable and interpretable network estimation. In the absence of formal power criteria, researchers are encouraged to rely on sample size adequacy and to verify the robustness of the estimated network through stability and accuracy analyses. In the present study, this was supported by the high correlation stability coefficient (CS = 0.672) and the overall accuracy of edge weight estimates.

The original research from which this data was derived was approved by the Ethical Committee “Lazio I,” Regione Lazio, with Protocol No. 1058/03.06.2019. In this study, both the Declaration of Helsinki and other relevant institutional ethical standards were followed [40].

### Measures

#### Structured Clinical Interview for DSM-5 for Personality Disorders (SCID-5-PD)

Personality disorder diagnoses were established using the *Structured Clinical Interview for DSM-5 Personality Disorders* (SCID-5-PD; [41]. Inter-rater reliability was assessed on 25 SCID-5-PD interviews that were independently double-rated. Dimensional trait scores demonstrated excellent agreement, with ICCs (two-way mixed, absolute agreement) ranging from .88 to .99 (*M* = .93). Categorical diagnoses also showed strong reliability, with a mean Cohen’s *κ* of .89.

#### Personality Inventory for DSM-5 (PID-5)

The Personality Inventory for DSM-5 (PID-5; [31]) is a 220-item self-report questionnaire designed to measure the expression of 25 maladaptive personality trait facets according to Criterion B of the Alternative Model for Personality Disorders (AMPD) in the DSM-5. The 25 maladaptive traits are grouped within five high-order domains: (1) Negative Affectivity (tendency toward emotional instability, anxiety, and dysregulation of mood); (2) Detachment (social withdrawal, anhedonia, and interpersonal disengagement); (3) Antagonism (hostility, manipulativeness, and grandiosity); (4) Disinhibition (impulsivity and difficulties in planning or self-control), and (5) Psychoticism (eccentricity, perceptual dysregulation, and unusual beliefs). Respondents rate each statement on a four-point Likert scale ranging from 0 (*very false or often false*) to 3 (*very true or often true*). Higher mean scores reflect greater maladaptive expression of a given personality trait or domain. In the present sample, the Negative Affectivity domain presented a McDonald’s *ω* of .86, Detachment showed an *ω* of .81, Antagonism an *ω* of .91, and Disinhibition an *ω* of .84, and Psychoticism an *ω* of .80. Overall, these coefficients indicate good to excellent reliability across all PID-5 domains.

#### Symptom Checklist-90-Revised (SCL-90-R)

The Symptom Checklist-90-Revised (SCL-90-R; [42]) is a 90-item self-report inventory that assesses a wide range of psychological symptoms in clinical and medical populations. It measures current (state) symptomatology across nine primary symptom dimensions and provides an overall index of psychological distress, the Global Severity Index (GSI). For the purposes of this study, only the Depression subscale was used. In the present sample, the Depression subscale demonstrated good internal consistency, with a McDonald’s *ω* = .87.

#### Metacognition Assessment Interview (MAI)

The Metacognition Assessment Interview (MAI; [11, 43]) is a semi-structured interview aimed at evaluating metacognitive functioning. It is based upon a narrative task in which participants are asked to recount a personally meaningful autobiographical event that has happened within the last six months and has involved at least one other person. This allows the examiner to evaluate the individual’s ability to understand and reflect upon their own and others’ mental states.

After the narrative, participants respond to a set of structured questions that probe four metacognitive subfunctions: (1) monitoring: the ability to recognize and label components of mental states, such as emotions, thoughts, motives, and desires; (2) integration: the capacity to maintain a coherent and unified sense of self across varying experiences and contexts, while recognizing personal change and continuity over time; (3) differentiation: the ability to acknowledge the subjective nature of one’s own thoughts and to distinguish them from external reality, allowing cognitive flexibility; (4) decentration: the capacity to adopt another person’s perspective and infer their mental states; individuals with low decentration tend to interpret experiences solely from their own viewpoint. In the present research, the Monitoring subfunction showed a McDonald’s *ω* of .82, Differentiation an *ω* of .78, Decentration an *ω* of .90, and Integration an *ω* of .85. Overall, these values indicate good to excellent internal consistency across all metacognitive domains.

#### Pathological Narcissism Inventory (PNI)

The Pathological Narcissism Inventory (PNI; [44, 45]) is a 52-item self-report inventory rated on a six-point Likert scale, ranging from 0 (*“Does not describe me at all”*) to 5 (*“Describes me perfectly”*). The instrument comprises seven subscales: Exploitative, Grandiose Fantasy, Self-Sacrificing Self-Enhancement, Contingent Self-Esteem, Devaluing the Self, Entitlement Rage, and Hiding the Self. The first three subscales compose the Grandiosity dimension, whereas the latter four form the Vulnerability dimension. In the present study, only the total scores for the Grandiosity and Vulnerability dimensions were used in the analyses. In the current sample, McDonald’s *ω* values ranged from .82 (Vulnerability) to .90 (Grandiosity), indicating good internal consistency.

#### Inventory of Interpersonal Problems (IIP)

The Inventory of Interpersonal Problems (IIP; [46]) is a 47-item self-report measure assessing difficulties in interpersonal functioning. It contains five subscales that evaluate common maladaptive patterns in relationships: (1) Interpersonal Sensitivity, which reflects excessive concern about others’ evaluations and a tendency toward submissiveness and social anxiety; (2) Interpersonal Ambivalence reflects ambivalence concerning wanting to be close to and distant from others; (3) Aggression, represents hostile, controlling, or oppositional interpersonal behaviors; (4) Need for Social Approval describes an excessive need to be liked and accepted by others, often at the expense of one’s own needs; and (5) Lack of Sociability refers to avoidance of social interactions and a preference for isolation or emotional distance.

Together, these dimensions provide a comprehensive profile of interpersonal functioning and capture the maladaptive relational patterns that may contribute to psychological distress and personality pathology. In the present sample, the Interpersonal Sensitivity subscale showed a McDonald’s *ω* of .84, Interpersonal Ambivalence demonstrated an *ω* of .78, Aggression an *ω* of .92, Need for Social Approval an *ω* of .88, and Lack of Sociability an *ω* of .94. Overall, these coefficients indicate good to excellent reliability across all IIP subscales.

### Statistical analysis

Prior to network estimation, all variables were inspected for missing data and distributional properties. As previously described, participants with incomplete data (*n* = 30) were excluded from the analysis to avoid imputation procedures. All variables were treated as continuous and were standardized to ensure comparability across measures with different scales. No extreme outliers were detected. Given the robustness of regularized partial correlation networks, no additional transformations were applied.

Network analysis (NA) was performed using R [47] with the qgraph and bootnet packages [36, 48]. The network structure was estimated through a Gaussian Markov Random Field model with Least Absolute Shrinkage and Selection Operator (LASSO) regularization. This approach retains only the strongest and most reliable associations while reducing the likelihood of spurious connections [49]. The use of Extended Bayesian Information Criterion (EBIC) glasso regularization, with a tuning parameter value of 0.3, inherently addresses potential multicollinearity by shrinking redundant associations and setting small edge weights to zero [50]. Network sparsity was examined to ensure an appropriate balance between model parsimony and information retention.

Network estimation was done using the estimate Network function from the bootnet package [36]. To study the structural importance of each variable, node centrality indices were calculated by means of the centrality Plot function in qgraph. Centrality measures included betweenness (the extent to which a node lies on the shortest paths connecting other nodes), closeness (how closely a node is connected to all other nodes), strength (the sum of absolute edge weights directly connected to a node), and expected influence (the sum of all weighted connections, accounting for the sign of the edges).

Given that betweenness and closeness can often be hard to interpret in psychological networks [51], expected influence was chosen as the main index of node centrality, as it is a more stable and meaningful indicator of the influence of a node [36].

The correlation stability (CS) coefficient was used to evaluate the reliability of centrality estimates [36]. This coefficient provides the maximum proportion of the sample that can be dropped with at least a 0.7 correlation remaining between recalculated and original centrality indices. Values above 0.25 are generally considered acceptable for stable networks. CS coefficients were computed via case-drop bootstrapping (*n* = 2000). In addition, the accuracy of edge weights was assessed using non-parametric bootstrapping with 2000 samples, generating confidence intervals for each edge. Both bootstrap procedures were implemented through the bootnet function.

Finally, graphical representations of the network display the strength of associations through edge thickness: thicker edges indicate stronger relationships between variables. Edges in the network represent regularized partial correlations between variables, controlling for all other nodes. As such, they reflect undirected and non-causal associations, and should not be interpreted as evidence of directional or mechanistic relationships.

## Results

### Demographic and clinical characteristics of the sample

Table 1 presents the socio-demographic characteristics of the sample (*N* = 287). The mean age of participants was 34.9 years (SD = 11.0). Gender distribution was balanced, with 49.1% identifying as male and 50.9% as female. Most participants in the sample were single (61.3%), followed by married (29.6%), divorced (8.4%), and widowed (0.7%). Regarding education, over half of the participants had completed high school (53.3%), and 40.8% held a master’s degree. Only a few reported having middle school (4.5%) and elementary school education (1.4%). In terms of employment status, the largest group was employed (42.5%), followed by students (24.0%). Practitioners represented 18.1% of the sample, while smaller percentages identified as unemployed (8.7%), retired (3.5%), or engaged in unpaid activities (3.2%).

**Table 1.** Socio-demographic features of the sample Table 1. long description.

		*N* = 287	
		Fr	%
Mean age (years)^a^		34.9	(11.0)
Gender	Male	141	(49.1)
	Female	146	(50.9)
Civil status	Married	85	(29.6)
	Divorced	24	(8.4)
	Widow	2	(0.7)
	Single	176	(61.3)
Education	Elementary school	4	(1.4)
	Middle school	13	(4.5)
	High school	153	(53.3)
	Master	117	(40.8)
Employment	Employed	122	(42.5)
	Unemployed	25	(8.7)
	On pension	10	(3.5)
	Student	69	(24.0)
	Unpaid activity	9	(3.2)
	Practitioner	52	(18.1)

a Data are expressed as means and (standard deviation).

### Network analysis in patients with NPD

**Figure 2.**
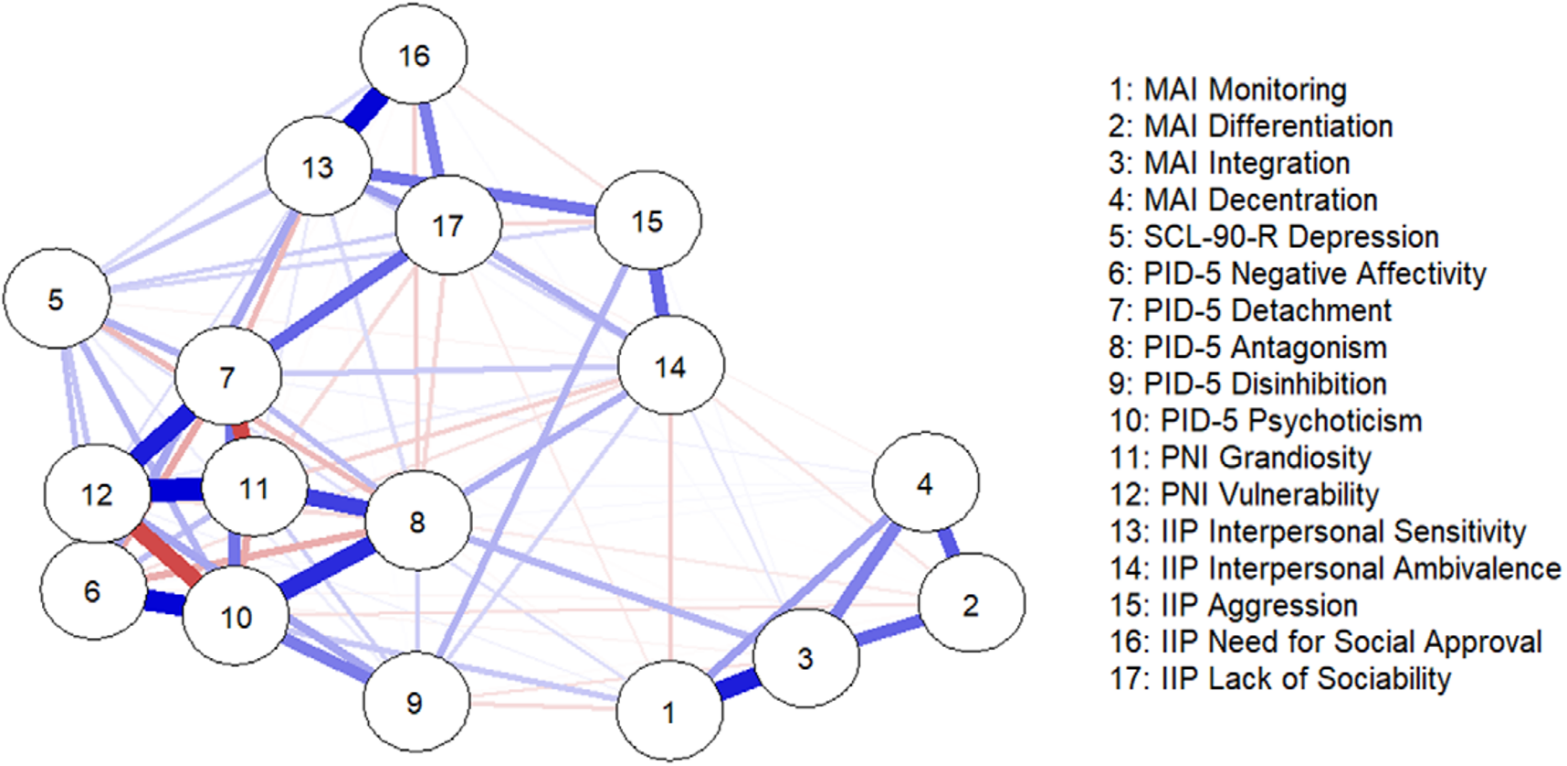
The network structure estimated from the graphical EBIC-LASSO in patients with NPD. Nodes represent psychological variables and are numbered for clarity: (1) MAI Monitoring, (2) MAI Differentiation, (3) MAI Integration, (4) MAI Decentration, (5) SCL-90-R Depression, (6) PID-5 Negative Affectivity, (7) PID-5 Detachment, (8) PID-5 Antagonism, (9) PID-5 Disinhibition, (10) PID-5 Psychoticism, (11) PNI Grandiosity, (12) PNI Vulnerability, (13) IIP Interpersonal Sensitivity, (14) IIP Interpersonal Ambivalence, (15) IIP Aggression, (16) IIP Need for Social Approval, (17) IIP Lack of Sociability. Blue lines represent positive correlations, and red lines represent negative correlations. Thicker edges represent stronger correlations. Centrality analysis indicated that narcissistic vulnerability (node 12), interpersonal sensitivity (node 13), and metacognitive integration (node 3) were the most influential nodes in the network. Figure 2. long description.

Figure 2 presents the network analysis. The resulting sparsity (0.346) fell within the typical range reported in psychological network studies, suggesting adequate regularization without over- or underfitting. Node labels in Figure 2 correspond to the following variables: (1) MAI Monitoring, (2) MAI Differentiation, (3) MAI Integration, (4) MAI Decentration, (5) SCL-90-R Depression, (6) PID-5 Negative Affectivity, (7) PID-5 Detachment, (8) PID-5 Antagonism, (9) PID-5 Disinhibition, (10) PID-5 Psychoticism, (11) PNI Grandiosity, (12) PNI Vulnerability, (13) IIP Interpersonal Sensitivity, (14) IIP Interpersonal Ambivalence, (15) IIP Aggression, (16) IIP Need for Social Approval, and (17) IIP Lack of Sociability. The most central of the nodes, in terms of expected influence (EI), were Vulnerability (EI = 2.15), Interpersonal Sensitivity (EI = 1.99), and Metacognitive Integration (EI = 1.06). These variables emerged as the most structurally central components of the network, indicating that they are highly interconnected with other psychological domains and may play an important role in organizing the overall configuration of narcissistic pathology. Conversely, only two variables had a clearly negative influence: Grandiosity (EI = –1.42) and Metacognitive Differentiation (EI = –1.13). The strongly negative values of these variables suggest that they are peripheral or balancing elements in the network and contribute less to overall system activation than the central nodes identified above.

Centrality results for strength, betweenness, and closeness are presented in the supplemental material (Supplementary Figure S1). The expected influence correlation stability coefficient (CS) was 0.672, considerably greater than the 0.25 threshold for a high interpretability of node centrality.

Among the strongest partial correlations observed across different measures (Table 2) were the associations between PID-5 Detachment and PNI Vulnerability (*r* = .45) and between PID-5 Negative Affectivity and PNI Vulnerability (*r* = .47), indicating that interpersonal withdrawal and emotional instability are closely linked to heightened narcissistic vulnerability. Importantly, a strong positive association was also found between PID-5 Antagonism and PNI Grandiosity (*r* = .38), pointing to the convergence between antagonistic personality features and grandiose expressions of pathological narcissism. Finally, a smaller but meaningful link emerged between IIP Lack of Sociability and PID-5 Detachment (*r* = .31), underscoring the alignment of interpersonal avoidance with maladaptive personality traits related to social disengagement.

**Table 2.** Partial correlation matrix from network analysis in patients with Narcissistic Personality Disorder (*N* = 287) Table 2. long description.

	1	2	3	4	5	6	7	8	9	10	11	12	13	14	15	16	17
1. MAI Monitoring	–																
2. MAI Differentiation	0.07	–															
3. MAI Integration	0.45	0.31	–														
4. MAI Decentration	0.19	0.33	0.25	–													
5. SCL-90-R Depression	0.06	0.00	−0.01	0.00	–												
6. PID-5 Negative Affectivity	0.11	−0.02	−0.03	0.01	0.13	–											
7. PID-5 Detachment	−0.01	0.00	0.00	0.03	0.15	−0.17	–										
8. PID-5 Antagonism	−0.02	−0.04	0.15	0.01	−0.14	−0.16	0.14	–									
9. PID-5 Disinhibition	−0.07	−0.01	−0.06	0.00	0.03	0.00	0.00	0.10	–								
10. PID-5 Psychoticism	−0.03	−0.07	0.00	0.00	0.15	0.50	0.28	0.42	0.26	–							
11. PNI Grandiosity	0.00	0.00	0.00	0.00	0.00	0.13	−0.16	0.38	0.10	−0.17	–						
12. PNI Vulnerability	0.03	0.00	0.00	0.02	0.12	0.47	0.45	−0.07	0.18	−0.36	0.51	–					
13. IIP Interpersonal Sensitivity	0.00	0.01	0.00	0.00	0.11	0.18	−0.14	0.07	0.00	0.00	0.04	0.07	–				
14. IIP Interpersonal Ambivalence	−0.09	−0.05	0.05	0.00	−0.02	−0.06	0.11	0.16	0.08	−0.02	−0.09	0.03	0.09	–			
15. IIP Aggression	0.00	0.00	0.02	0.00	0.08	0.00	0.00	0.00	0.15	0.00	0.00	0.00	0.28	0.30	–		
16. IIP Need for Social Approval	0.00	0.00	0.01	0.00	0.07	0.00	−0.01	−0.10	−0.01	0.00	−0.01	0.00	0.50	0.00	−0.05	–	
17. IIP Lack of Sociability	−0.04	0.00	0.00	−0.03	0.10	0.00	0.31	−0.08	0.00	0.00	−0.07	0.00	0.20	0.16	−0.08	0.26	–

Abbreviations: IIP, Inventory of Interpersonal Problems; MAI, Metacognition Assessment Interview; PID-5, Personality Inventory for DSM-5; PNI, Pathological Narcissism Inventory; SCL-90-R, Symptom Checklist-90-Revised.

Overall, the supplementary analyses indicate that the estimated network is adequately stable and interpretable. The case-dropping bootstrap showed acceptable stability of centrality indices (Supplementary Figure S2), the bootstrapped confidence intervals for edge weights were generally narrow (Supplementary Figure S3), and centrality estimates demonstrated sufficient precision, supporting the robustness of the reported network structure and node importance (Supplementary Figure S4).

## Discussion

The present study explored how metacognition, narcissistic dimensions, maladaptive personality traits, interpersonal functioning, and depressive symptoms interact in a sample diagnosed with NPD. By modeling these domains within a single network, we aimed to capture the dynamic interplay that characterizes narcissistic pathology. The resulting structure underlines several psychologically meaningful patterns that shed light on both the vulnerabilities and potential regulatory mechanisms embedded in the narcissistic system.

It is important to note that the present findings reflect contemporaneous associations among variables rather than temporal or causal processes. In this context, the term “dynamic” is used to describe the patterned interdependence and mutual connectivity among psychological domains within the network. Interpretations regarding regulatory functions or amplifying processes are therefore grounded in established theory, prior empirical research, and clinical observation, rather than direct causal inference from the cross-sectional network.

One of the central findings of this study is the prominent centrality of narcissistic vulnerability within the network. Its high expected influence suggests that narcissistic vulnerability constitutes the psychological source of distress in NPD, shaping and being shaped by a wide array of emotional, cognitive, and interpersonal processes. This dimension denotes a fragile and fluctuating self-structure, characterized by shame, feelings of inadequacy, susceptibility to criticism, and the perception of interpersonal environments as threatening or rejecting [5, 52]. Centrality of narcissistic vulnerability is in line with state-of-the-art theoretical models that consider vulnerability, rather than grandiosity, as the most reactive and clinically onerous form of narcissistic functioning [53–55].

Vulnerability appears to occupy a structurally central position within the network that is consistent with theoretical and clinical models in which vulnerable self-states are closely intertwined with maladaptive self-regulatory strategies, such as withdrawal, hostility, or compensatory grandiosity. This interpretation is consistent with recent intensive longitudinal evidence showing that narcissistic vulnerability represents a proximal source of emotional distress. Using EMA, Edershile and colleagues demonstrated that vulnerability and contingent self-esteem are associated with higher momentary negative affect and amplify the coupling between negative affect and self-esteem, highlighting vulnerability as a core driver of distress across time [56]. Although the present cross-sectional data do not permit causal inferences, this configuration aligns with clinical observations suggesting that vulnerability-related affects and beliefs are tightly linked to these regulatory responses [57–60].

**Figure 3.**
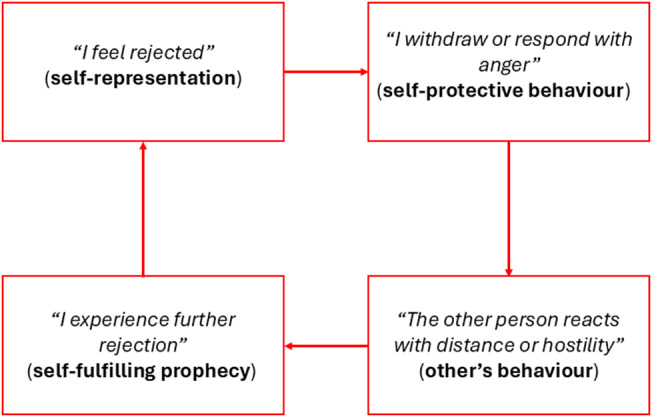
Interpersonal cycle of rejection in Narcissistic Personality Disorder. Figure 3. long description.

Interpersonal sensitivity also emerged as a highly influential node, highlighting its central contribution to the maintenance of narcissistic vulnerability. This construct captures the pervasive attentiveness to others’ evaluations, fear of rejection, and difficulty tolerating interpersonal ambiguity [61, 62]. Its strong centrality indicates that interpersonal sensitivity occupies a highly central position within the network and is strongly connected to narcissistic vulnerability. This centrality is consistent with the idea that heightened sensitivity to evaluation is closely associated with intensified experiences of vulnerability within interpersonal contexts. While causality cannot be inferred, the pattern mirrors clinically described self-reinforcing interpersonal cycles, commonly referred to as self-fulfilling prophecies or vicious circles [63]. This self-reinforcing loop illustrates how interpersonal sensitivity can maintain and amplify the fragile self-states characteristic of narcissistic vulnerability (Figure 3), and indicates that interpersonal sensitivity may represent an important target for intervention [64, 65].

Beyond the dimensional organization proposed by the AMPD, the network’s strongest partial correlations provide further insight into the specific dispositional pathways that differentiate grandiose and vulnerable narcissistic functioning. The robust associations observed between PID-5 Negative Affectivity and Vulnerability, as well as between PID-5 Detachment and Vulnerability, underscore the centrality of emotional instability, shame reactivity, and interpersonal withdrawal in the vulnerable phenotype. These links support the view that narcissistic vulnerability is organized around an internalizing affective core in which dysregulated negative self-conscious emotions combine with social inhibition and avoidance, producing a self–other dynamic marked by hypersensitivity, fear of rejection, and the tendency to retreat from interpersonal engagement [3, 5, 59]. In contrast, the strong positive association between PID-5 Antagonism and Grandiosity highlights the interpersonal dominance, entitlement, and exploitativeness that characterize the grandiose phenotype. This pattern is consistent with extensive evidence showing that antagonistic traits such as manipulativeness, arrogance, and callousness constitute the dispositional backbone of narcissistic grandiosity [32, 33]. Together, these findings suggest that the two primary narcissistic dimensions draw upon distinct but complementary personality systems: vulnerability is embedded within internalizing distress and relational withdrawal, whereas grandiosity is embedded within externalizing, antagonistic interpersonal strategies. This dual structure may help explain the oscillation many individuals exhibit between defensive superiority and painful self-doubt, as shifts in interpersonal context may differentially activate these dispositional networks [27, 66, 67]. From an evolutionary perspective, these distinct patterns may reflect different strategies related to social rank regulation and interpersonal competition, with vulnerability associated with threat sensitivity and withdrawal, and grandiosity associated with dominance and status-seeking behaviors. When these strategies become rigid or dysregulated, they may contribute to maladaptive interpersonal and affective functioning [68].

A third key finding relates to metacognitive functioning and especially to metacognitive integration, since it came out as a relatively central node within the wider system. Although past literature often highlights impairments in basic monitoring of mental states and decentration among individuals with narcissistic pathology [21, 69–71], the present findings indicate that integration ability is a central node along with vulnerability and interpersonal sensitivity, suggesting it may serve an important function in the narcissistic network. Metacognitive integration refers to the ability to construct a coherent and temporally continuous understanding of one’s internal experiences, even when emotions or thoughts fluctuate [72]. Individuals with higher integrative capacities are more likely to retain a cohesive identity, reduce internal fragmentation, and contextualize emotional reactions within a wider personal perspective. This facilitates emotional regulation and fosters more stable and flexible interpersonal functioning. In our network, metacognitive integration bridges narcissistic vulnerability and interpersonal sensitivity with more adaptive elements of psychological functioning (i.e., metacognition). This pattern is consistent with the idea that higher integrative capacities may be related to a greater ability to process emotionally and interpersonally complex experiences.

One of the core principles of MIT involves helping patients recognize their most intimate desires and emotional needs, particularly those that are suppressed when they conflict with grandiose self-expectations. MIT aims to foster contact with these vulnerable states and integrate them into a coherent self-experience, rather than allowing the individual to remain trapped in the pursuit of an idealized self [73, 74]. Only after strengthening self-awareness and metacognitive integration is it possible to work effectively on understanding the minds of others and promoting decentration [10, 73, 75].

These results are in line with theoretical and clinical models emphasizing that the development of metacognitive integration may provide a decisive contribution to the stabilization of identity and to functional interpersonal patterns [19, 34]. They further suggest that treatment should focus on enhancing integrative capabilities as a counterbalance to NPD vulnerabilities. A broader interpretation of these findings can be framed within psychotherapeutic approaches to personality disorders. Dimensional and integrative models, such as Good Psychiatric Management, conceptualize narcissistic pathology as characterized by difficulties in self-esteem regulation, with patients oscillating between states of threatened self-worth and attempts to restore a coherent and often idealized sense of self [76]. In this context, metacognitive integration may represent a more specific mechanism through which individuals are able to understand, organize, and regulate these fluctuating self-states. In addition, recent evidence suggests that mindfulness- and compassion-based approaches may be particularly relevant for individuals with narcissistic traits, especially in relation to the regulation of shame and self-critical processes [77]. These approaches may complement metacognitive interventions by fostering non-judgmental awareness and more adaptive responses to perceived interpersonal threat.

Taken together, the network configuration conveys a dynamic tension between vulnerability and regulation. On the one hand, the vulnerable–interpersonal subsystem, driven by narcissistic vulnerability and interpersonal sensitivity, is associated with heightened emotional reactivity and instability in self-structure. On the other hand, metacognitive integration is positioned within the network in a way that is consistent with the idea that difficulties in understanding and integrating internal states may be linked to increased vulnerability, whereas stronger integrative capacities may be related to more adaptive self and interpersonal functioning.

The results are consistent with the theoretical underpinning of MIT. According to the latter, narcissistic vulnerability consists of the manifestation of a fragile, easily disrupted self-structure, sensitive to shameful feelings and interpersonal devaluation. Interpersonal sensitivity is understood as a trigger that reactivates interpersonal schemas of threat and judgment, thereby setting in motion cycles of avoidance, anger, or compensatory grandiosity [10, 78]. Within this model, the enhancement of metacognitive integration is considered a central therapeutic goal. Enhancing the patient’s ability to understand, organize, and link internal states over time fosters a more coherent and stable self-narrative. As integrative capacities strengthen, patients become better equipped to manage shame, regulate emotional responses, and adopt more flexible and compassionate interpersonal strategies. The present network provides empirical support for this clinical formulation, suggesting that interventions aimed at strengthening metacognitive integration may shift patients from defensive and reactive functioning toward more authentic, stable, and adaptive forms of self-experience.

### Strengths and limitations

This study has several notable strengths. It is, to the best of our knowledge, the first to examine the combined network of metacognition, narcissistic dimensions, maladaptive personality traits, interpersonal difficulties, and depressive symptoms specifically in a large clinically diagnosed NPD sample. The use of a robust network analytic framework, combined with rigorous bootstrapped accuracy and stability indices, yields a fine-grained depiction of important interconnections among key psychological processes. Furthermore, the investigation of metacognitive capacities, an understudied yet theoretically central domain, offers novel insights into possible mechanisms that sustain narcissistic pathology and identifies potential targets for intervention. This may be particularly useful for the development of empirically validated treatments, which remain relatively scarce for NPD compared to borderline personality disorder, despite the high prevalence of NPD in outpatient settings [79–81].

On the other hand, several limitations should also be considered. First, the cross-sectional design precludes any causal or temporal inference regarding the directionality of associations within the network. Although the observed structure is consistent with theoretical and clinical models, interpretations in terms of influence, regulation, or feedback processes should be considered as theory-informed rather than empirically causal. Longitudinal or intensive repeated-measures designs would be necessary to clarify temporal dynamics and directional pathways among variables. Second, the sample was diagnostically homogeneous and it was drawn from a single specialized outpatient clinic for personality disorders, which may limit the generalizability of findings to more heterogeneous or community-based populations, where comorbidity patterns and symptom configurations may differ. In addition, the relatively balanced sex distribution observed in the present sample differs somewhat from community-based findings indicating gender differences in narcissistic traits, although reported effect sizes are generally modest rather than large [82]. This may reflect characteristics of the specialized referral setting and associated case mix, and should therefore be considered when generalizing the present results. Third, potential conceptual and measurement overlap between constructs should be considered. For example, narcissistic vulnerability and negative affectivity both capture aspects of emotional dysregulation and distress, which may inflate their associations within the network. Although the use of partial correlation networks aims to isolate unique relationships among variables, shared variance at the construct level cannot be entirely ruled out and may influence the observed structure. Therefore, the prominence of certain nodes may partially reflect overlapping psychological domains rather than entirely distinct processes. Future studies could benefit from formally assessing construct redundancy and exploring clustering or dimension reduction approaches. Finally, it is important to acknowledge that network estimation is sensitive to sample size, variable selection, and regularization parameters. Although the present sample size was consistent with current recommendations and the network demonstrated good stability and accuracy (e.g., CS = 0.672), different modeling choices (e.g., alternative tuning parameters or node selections) could yield partially different network configurations. Therefore, replication in independent samples is warranted to confirm the robustness and generalizability of the present findings.

## Conclusion

This study provides new evidence that narcissistic vulnerability and interpersonal sensitivity form the source of psychological distress in NPD. These processes appear to drive emotional instability, hypersensitivity to evaluation, and maladaptive interpersonal cycles. In contrast, metacognitive integration emerged as a centrally positioned node within the network, suggesting that it may be closely associated with the organization of psychological processes in NPD. From a clinical perspective, these findings align closely with MIT. They emphasize the importance of helping patients strengthen their ability to recognize, link, and integrate their mental states, particularly when confronted with shame, rejection, or interpersonal threat. Reinforcing metacognitive integration may reduce fragmentation, support more stable self-regulation, and promote flexible interpersonal behaviors. Although cross-sectional, the study underscores the relevance of targeting vulnerability, interpersonal hypersensitivity, and metacognitive deficits as priority mechanisms of change. Future longitudinal research should examine whether improvements in integration mediate therapeutic gains in NPD.

## Supporting information

10.1192/j.eurpsy.2026.12226.sm001 Aloi et al. supplementary material Aloi et al. supplementary material

## Data Availability

The datasets generated and/or analyzed for the current study contain clinical data and are not publicly available due to the protection of participants’ rights to privacy and data protection. Further inquiries can be directed to the corresponding author.

## Supplementary material

The supplementary material for this article can be found at http://doi.org/10.1192/j.eurpsy.2026.12226.

## Long descriptions

### [Figure 1] Long description

The flowchart is divided into two main phases labeled on the left: Screening and Enrollment.

1. Screening Phase

At the top, a box states Patients assessed for eligibility n equals 518.

A vertical arrow points down to the next stage, while a horizontal arrow points right to an exclusion box.

The exclusion box contains two groups: Patients with other personality disorders than N P D n equals 137, and Patients with other psychiatric disorders than N P D n equals 64.

The vertical arrow leads to a box labeled Screened patients with N P D n equals 317.

2. Enrollment Phase

A vertical arrow points down from the screened patients box to the final inclusion box, while a horizontal arrow points right to an exclusion box.

The exclusion box states Patients did not complete the assessment n equals 30.

The final box at the bottom of the vertical path is labeled Patients included in the present study n equals 287.

Navigate back to [Figure 1].

### [Table 1] Long description

The table presents data for N = 287 participants across five main categories.

* Mean age: 34.9 years with a standard deviation of 11.0.

* Gender: Male F r 141 (49.1 percent); Female F r 146 (50.9 percent).

* Civil status: Married F r 85 (29.6 percent); Divorced F r 24 (8.4 percent); Widow F r 2 (0.7 percent); Single F r 176 (61.3 percent).

* Education: Elementary school F r 4 (1.4 percent); Middle school F r 13 (4.5 percent); High school F r 153 (53.3 percent); Master F r 117 (40.8 percent).

* Employment: Employed F r 122 (42.5 percent); Unemployed F r 25 (8.7 percent); On pension F r 10 (3.5 percent); Student F r 69 (24.0 percent); Unpaid activity F r 9 (3.2 percent); Practitioner F r 52 (18.1 percent).

Navigate back to [Table 1].

### [Figure 2] Long description

A network graph with 17 numbered nodes connected by varying thicknesses of blue and red edges.

* Central and Left Cluster: Node 12 P N I Vulnerability is a central hub with thick blue edges connecting to node 11 P N I Grandiosity, node 7 P I D 5 Detachment, and node 6 P I D 5 Negative Affectivity. A prominent red edge indicates a negative correlation between node 12 and node 10 P I D 5 Psychoticism. Node 8 P I D 5 Antagonism is also centrally located, showing strong blue connections to nodes 10 and 11.

* Top and Upper-Left Region: Node 5 S C L 90 R Depression is on the far left, connected by thin blue lines to the central cluster. Node 13 I I P Interpersonal Sensitivity and node 16 I I P Need for Social Approval are at the top, joined by a very thick blue edge. Node 17 I I P Lack of Sociability sits below them, connecting to node 7 and node 15 I I P Aggression.

* Right and Lower Region: Node 14 I I P Interpersonal Ambivalence acts as a bridge between the center and the right side. On the far right, a distinct triangle is formed by node 4 M A I Decentration, node 2 M A I Differentiation, and node 3 M A I Integration. Node 1 M A I Monitoring is at the bottom, connected most strongly to node 3.

Thicker blue lines represent the strongest positive correlations, notably between nodes 12 and 11, 13 and 16, and 3 and 1.

Navigate back to [Figure 2].

### [Table 2] Long description

The table presents a lower triangular matrix of partial correlations for 17 variables. The variables are grouped by assessment tool: M A I (1 to 4), S C L 90 R (5), P I D 5 (6 to 10), P N I (11 to 12), and I I P (13 to 17).

Key correlations include:

* Strongest positive within-group correlations: M A I Monitoring and Integration (0.45), P I D 5 Negative Affectivity and Psychoticism (0.50), P N I Grandiosity and Vulnerability (0.51), and I I P Interpersonal Sensitivity and Need for Social Approval (0.50).

* Strongest cross-group correlations: P I D 5 Negative Affectivity and P N I Vulnerability (0.47), P I D 5 Detachment and P N I Vulnerability (0.45), and P I D 5 Antagonism and P I D 5 Psychoticism (0.42).

* Notable negative correlations: P I D 5 Psychoticism and P N I Vulnerability (-0.36), and P I D 5 Psychoticism and P N I Grandiosity (-0.17).

Variable List:

1. M A I Monitoring

2. M A I Differentiation

3. M A I Integration

4. M A I Decentration

5. S C L 90 R Depression

6. P I D 5 Negative Affectivity

7. P I D 5 Detachment

8. P I D 5 Antagonism

9. P I D 5 Disinhibition

10. P I D 5 Psychoticism

11. P N I Grandiosity

12. P N I Vulnerability

13. I I P Interpersonal Sensitivity

14. I I P Interpersonal Ambivalence

15. I I P Aggression

16. I I P Need for Social Approval

17. I I P Lack of Sociability

Navigate back to [Table 2].

### [Figure 3] Long description

The flowchart consists of four red rectangular boxes connected by red arrows in a continuous clockwise loop.

* The top-left box contains the text: I feel rejected, which is categorized as self-representation.

* An arrow points right to the top-right box, which contains the text: I withdraw or respond with anger, categorized as self-protective behaviour.

* An arrow points down to the bottom-right box, which contains the text: The other person reacts with distance or hostility, categorized as other’s behaviour.

* An arrow points left to the bottom-left box, which contains the text: I experience further rejection, categorized as self-fulfilling prophecy.

* A final arrow points upward from the bottom-left box back to the top-left box, completing the cycle.

Navigate back to [Figure 3].
