# Supplementary material for: The interplay among narcissistic vulnerability, interpersonal sensitivity, and metacognitive integration: A network analysis approach
Source: Eur Psychiatry. 2026 Jun 16;69(1):e64. doi: 10.1192/j.eurpsy.2026.12226 (PMC13276720; doi:10.1192/j.eurpsy.2026.12226)
Supplement: Aloi et al. supplementary material [file S0924933826122263sup001.docx]

**Supplementary figures**

| **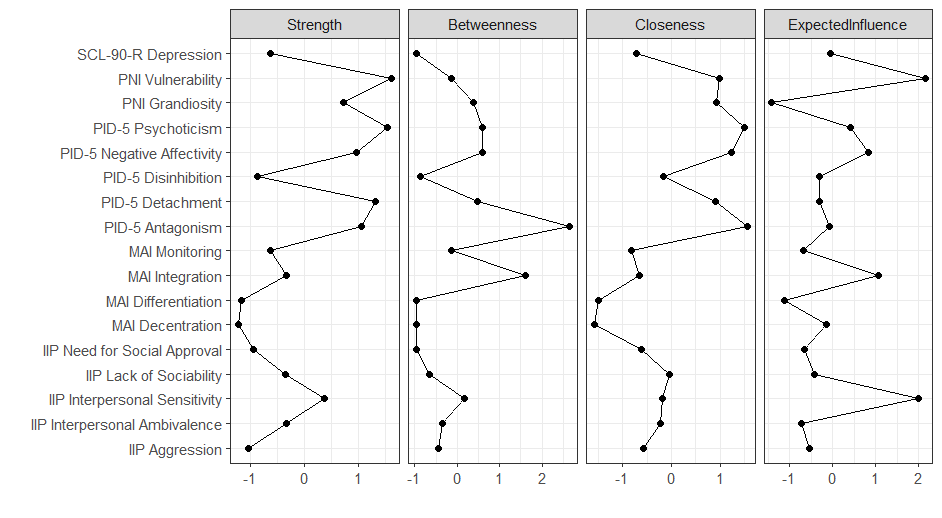** |
| --- |
| ***Figure S1****. Centrality indices of the estimated network in patients with NPD. Strength, Betweenness, Closeness, and Expected Influence values are displayed for each node included in the network model.* |

| 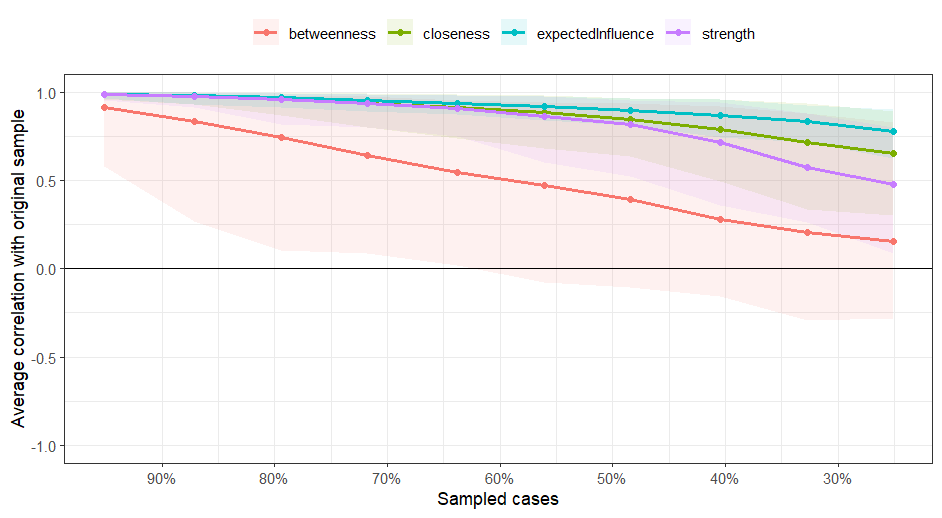 |
| --- |
| ***Figure S2****. Results of case-dropping subset bootstrap procedure to assess stability of network centrality indices. Average correlations between centrality indices of networks sampled with persons dropped and the original sample of person with NPD. Lines indicate the means and areas indicate the range from the 2.5^th^ quantile to the 97.5^th^ quantile.* |

| 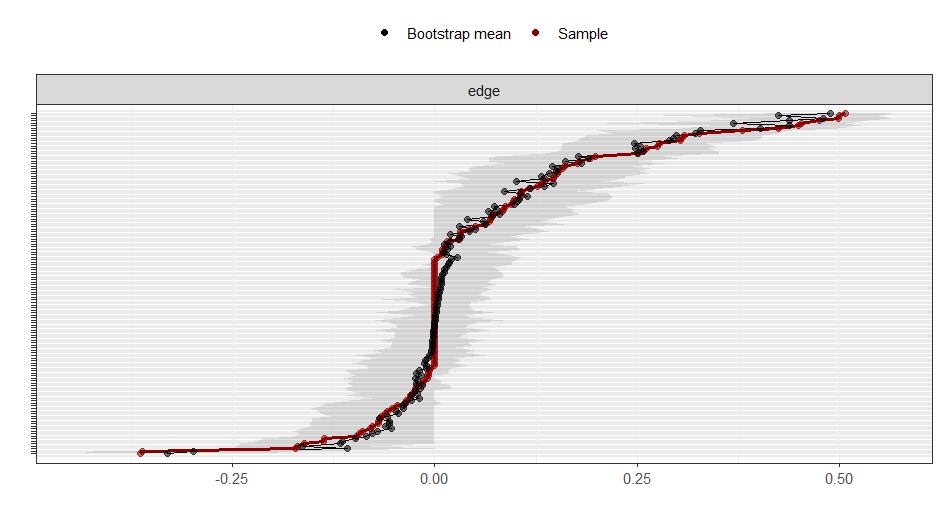 |
| --- |
| ***Figure S3****. Bootstrapped confidence intervals (#boots = 2000) for estimated edge-weights of network analysis.* |

| 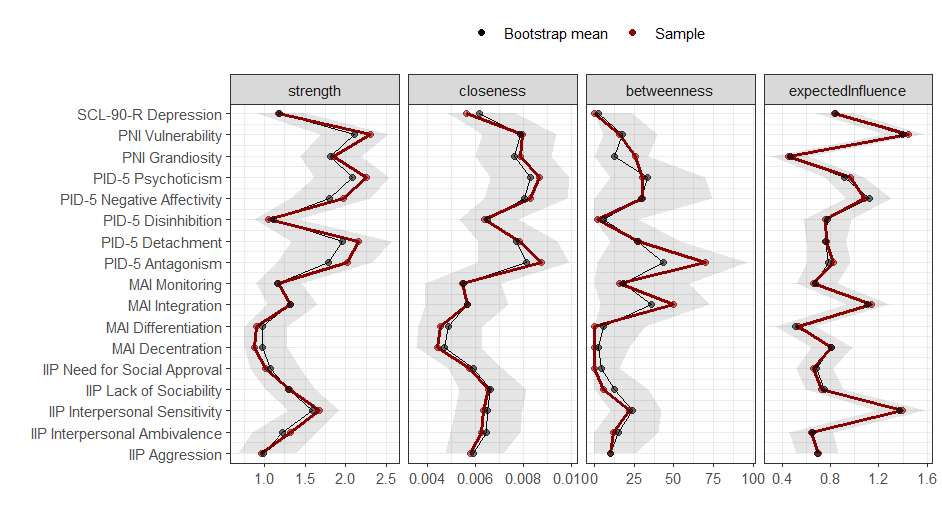 |
| --- |
| *Figure S4. Bootstrapped confidence intervals (#boots = 2000) for estimated centrality indices of network analysis.* |
